# Overcoming Tumor Hypoxia in Photodynamic Therapy: A Comprehensive Review of Oxygen-Delivery Carriers and Type I Photosensitizers

**DOI:** 10.3390/ijms27114748

**Published:** 2026-05-25

**Authors:** Dorota Bartusik-Aebisher, Izabela Rudy, Kacper Rogóż, Jakub Szpara, Aleksandra Kawczyk-Krupka, David Aebisher

**Affiliations:** 1 Department of Biochemistry and General Chemistry, Faculty of Medicine, Collegium Medicum, University of Rzeszów, 35-959 Rzeszów, Poland; 2 Student Scientific Club of Biochemists URCell, Department of Biochemistry and General Chemistry, Faculty of Medicine, Collegium Medicum, University of Rzeszów, 35-959 Rzeszów, Polandkr117626@stud.ur.edu.pl (K.R.);; 3 Department of Internal Diseases, Angiology and Physical Medicine, Center for Laser Diagnostics and Therapy, Faculty of Medical Sciences in Zabrze, Medical University of Silesia, 40-055 Katowice, Poland; 4 Department of Photomedicine and Physical Chemistry, Medical College, University of Rzeszów, 35-959 Rzeszów, Poland

**Keywords:** tumor hypoxia, perfluorocarbon oxygen carriers, photodynamic therapy, type I photosensitizers, nanomedicine

## Abstract

Hypoxia is one of the most important factors limiting the effectiveness of modern anticancer therapies, particularly photodynamic therapy (PDT). The hypoxia of the tumor microenvironment results from abnormal angiogenesis and the high metabolic demand of cancer cells, which leads to reduced oxygen availability necessary for generating reactive oxygen species (ROS). Consequently, conventional therapeutic approaches, mainly based on the type II PDT mechanism, show limited effectiveness under hypoxic conditions. In response to these limitations, strategies are being developed to increase oxygen availability within the tumor. Of particular importance are nanocarriers based on perfluorocarbons (PFCs), which, due to their high gas solubility, can effectively transport and release oxygen in the tumor microenvironment. Research indicates that the use of such systems leads to improved PDT efficiency by increasing the production of singlet oxygen and enhancing cancer cell damage. Parallelly, alternative approaches independent of high oxygen concentration, including type I photosensitizers, are being developed. Unlike classical type II mechanisms, they generate free radicals through electron transfer reactions, which allows effective action even under conditions of significant hypoxia. This approach significantly expands the possibilities of using PDT in the treatment of tumors with low oxygen levels. Current research directions focus on integrating various therapeutic strategies to achieve a synergistic effect. Hybrid systems combining oxygen delivery (e.g., using PFCs) with the use of type I photosensitizers and other treatment methods, such as chemotherapy or immunotherapy, show the greatest clinical potential. Such multifunctional approaches simultaneously allow improving tumor oxygenation and increasing the efficiency of ROS generation, which makes them a promising strategy for the future of anticancer therapies.

## 1. Introduction

Tumor hypoxia is defined as a state of reduced oxygen levels in tumor tissue, resulting from insufficient oxygen supply relative to the metabolic demand of the cells. This phenomenon is one of the key features of the microenvironment of solid tumors and is associated with a more aggressive tumor phenotype, resistance to treatment, and poorer clinical prognosis [1,2]. The tumor microenvironment is characterized by low partial pressure of oxygen (pO_2_), resulting from an abnormal, chaotic network of blood vessels and impaired perfusion. Rapid growth of cancer cells leads to inadequate oxygen supply, causing areas of chronic hypoxia. Additionally, the imbalance between oxygen delivery and consumption reinforces this unfavorable metabolic environment [3,4]. Oxygen plays a key role in many anticancer therapies, particularly in photodynamic therapy (PDT), in which a light-activated photosensitizer transfers energy to oxygen molecules, leading to the formation of reactive oxygen species (ROS) capable of destroying cancer cells. The effectiveness of this process is directly dependent on the availability of oxygen in the tumor tissue [5]. Hypoxia significantly limits the effectiveness of oxygen-dependent therapies, such as PDT, because reduced oxygen availability leads to decreased ROS production. As a result, the effectiveness of cancer cell destruction is significantly reduced, which constitutes one of the main clinical barriers to the application of these methods [6,7]. Due to the limitations resulting from hypoxia, the aim of this article is to review contemporary strategies aimed at increasing the effectiveness of anticancer therapies by (1) improving the delivery of oxygen to the tumor and (2) developing alternative mechanisms for generating reactive oxygen species that operate independently of its presence. These approaches include, among others, the use of oxygen nanocarriers and the modification of photosensitizer action mechanisms [5,6].

## 2. Mechanisms of Hypoxia Development in Tumors

### 2.1. Excessive Oxygen Consumption by Cancer Cells

Cancer cells are characterized by intensive metabolism, which results from their rapid proliferation and increased demand for energy and macromolecule biosynthesis. In response to limited oxygen availability, metabolic pathways are restructured, regulated, among others, by the transcription factor HIF-1, which coordinates the adaptation of cells to hypoxic conditions and supports their survival and further growth [7]. Additionally, the high metabolic activity of cancer cells leads to increased consumption of available oxygen, which deepens local hypoxia in the tumor microenvironment and promotes further adaptive changes that enable disease progression [8]. One of the key elements of the metabolic adaptation of tumors is the Warburg effect, which involves the preferential use of aerobic glycolysis instead of oxidative phosphorylation, even in the presence of oxygen. This phenomenon allows for rapid ATP production and provides precursors for the synthesis of biomolecules necessary for the intensive growth of cancer cells [9]. The Warburg effect is regulated by numerous factors, including HIF-1α, as well as enzymes of the glycolytic pathway, which allows cancer cells to maintain a high level of metabolism and adapt to the variable oxygen conditions in the tumor [8].

Immune and stromal cells exacerbate oxygen depletion in the tumor microenvironment (TME) by forming a high-demand, metabolically active ecosystem. Through intense glycolysis and aberrant angiogenesis, these non-cancerous populations consistently outpace the local oxygen supply, driving severe tissue hypoxia that suppresses antitumor immunity and promotes cancer progression. Rapidly expanding tumor masses create structural and functional vascular abnormalities, leading to an inherently restricted supply of oxygen. Tumor-associated macrophages (TAMs) frequently polarize into an immunosuppressive M2-like phenotype that consumes immense amounts of local oxygen. These alternatively activated macrophages significantly alter the TME by upregulating hypoxia-inducible factors (HIFs), which further adapt the local metabolism to survive without oxygen. TAMs release pro-angiogenic factors like vascular endothelial growth factor (VEGF), creating chaotic and leaky blood vessels that fail to deliver adequate oxygen.

Furthermore, tumor-associated neutrophils infiltrate the TME and undergo extensive respiratory bursts, consuming significant localized concentrations of oxygen. Myeloid-derived suppressor cells (MDSCs) heavily infiltrate hypoxic regions and exhibit high glycolytic rates, acting as metabolic sinks that deplete the TME of available oxygen.

Regulatory T cells (Tregs) also contribute to metabolic stress by reprogramming their own metabolism toward glycolysis, worsening local nutrient and oxygen deprivation. Stromal cells, specifically cancer-associated fibroblasts (CAFs), are major orchestrators of this microenvironmental oxygen depletion. CAFs exhibit an augmented glycolytic metabolism—known as the Warburg effect—even in the presence of oxygen, thereby converting abundant glucose into lactate. This hyper-glycolytic state of CAFs necessitates continuous oxygen consumption for energy production, stealing crucial resources from surrounding antitumor immune cells.

CAFs secrete cytokines such as transforming growth factor-beta (TGF-beta), which physically alter the extracellular matrix to restrict oxygen diffusion. The dense fibrotic stroma generated by CAFs physically compresses tumor blood vessels, effectively shutting down the perfusion of oxygenated blood. Because CAFs are highly sensitive to hypoxia, the initial oxygen depletion triggers a positive feedback loop where CAFs further modify the stroma to worsen the hypoxic condition. Endothelial cells within the TME are hijacked to form abnormal, leaky, and disorganized vascular networks that are incapable of proper systemic oxygen exchange. Mesenchymal stem cells (MSCs) recruited to the TME differentiate into additional stromal components, adding to the cumulative cellular mass and increasing baseline tissue oxygen demand.

The collective metabolic activity of these interacting immune and stromal cells overwhelms the compromised microvasculature, dropping oxygen partial pressure to critically low levels. This severe localized hypoxia subsequently impairs cytotoxic T lymphocyte function by disrupting their mitochondrial respiration and metabolic fitness. Natural killer NK cells are similarly deactivated in these depleted oxygen pockets, losing their ability to mount effective antitumor immune responses. Consequently, the escalating oxygen depletion fosters an immunosuppressive network that actively protects the tumor from immune-mediated destruction. Ultimately, the cooperative metabolic restructuring by immune and stromal cells establishes a harsh hypoxic niche that accelerates malignant progression and resistance to therapy [10,11].

Deficient tumor vasculature restricts the penetration and delivery of therapeutic molecules. Combining vascular normalization with type I photosensitizers provides a promising strategy. This approach improves the tumor microenvironment to boost treatment efficacy. Solid tumors grow rapidly and secrete an excess of pro-angiogenic factors (such as VEGF). This creates an abnormal, disorganized vascular network. These defective vessels are leaky and have a high interstitial fluid pressure, which creates an uneven fluid flow. Consequently, large therapeutic molecules—such as nanocarriers, antibodies, and standard type II photosensitizers (which rely heavily on oxygen)—struggle to penetrate deeply into the tumor. This poor delivery leaves tumor regions inadequately treated and hypoxic.

### 2.2. Biological Consequences

Hypoxia leads to the stabilization and activation of the hypoxia-inducible factor HIF-1α, which plays a central role in regulating the cellular response to oxygen deprivation. HIF-1α controls the expression of genes responsible for metabolic adaptation, cell survival, and their ability to invade and metastasize [8]. Activation of this factor is one of the key mechanisms that enable cancer cells to function under hypoxic conditions and maintain a high proliferation rate. HIF-1α stimulates the expression of pro-angiogenic factors, such as VEGF (vascular endothelial growth factor), which leads to the induction of angiogenesis. Newly formed blood vessels are intended to increase the supply of oxygen and nutrients to the tumor; however, they are often structurally and functionally abnormal, which perpetuates hypoxia [9]. This process represents a compensatory mechanism that simultaneously promotes further tumor development as well as its ability to invade and form metastases [8]. Hypoxia and the associated activation of HIF-1α contribute to the development of resistance to various forms of anticancer therapy, including radiotherapy and chemotherapy. Metabolic changes, such as the Warburg effect, and the activation of cellular survival pathways increase the ability of cancer cells to evade treatment-induced damage [12]. Furthermore, the hypoxic microenvironment favors the selection of more aggressive and resistant cell clones, which represents a significant clinical challenge and limits the effectiveness of available therapies [8].

As summarized in Table 1, the distinction between physical oxygen delivery and chemical type I photosensitization lies not only in their fundamental photochemical pathways but also in the architecture of the nanoparticle platforms utilized to deploy them (e.g., MOFs and liposomes for oxygen transport versus AIE dots and quantum dots for oxygen-independent radical generation).

### 2.3. Disturbances of Angiogenesis and Perfusion

**Figure 1:**
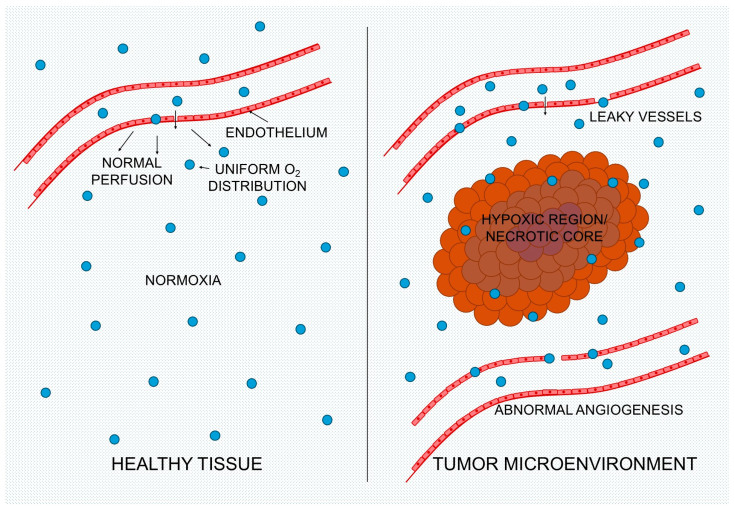
Schematic comparison of vascular architecture and oxygen distribution between healthy tissue and a solid tumor. Healthy tissue (**left**) is characterized by a hierarchical, organized, and tight vascular network that ensures uniform oxygen (O_2_) delivery and normoxic conditions. In contrast, tumor tissue (**right**) exhibits abnormal angiogenesis, resulting in chaotic, leaky, and distorted blood vessels. This dysfunctional vasculature, combined with high metabolic demand, creates a significant oxygen gradient. Areas distant from functional vessels transition from normoxia to hypoxia (blue/purple regions), eventually leading to a necrotic core in the center of the tumor where oxygen is completely depleted.

One of the main mechanisms leading to hypoxia in tumors is abnormal angiogenesis, resulting in the formation of blood vessels with disturbed structure and function. Unlike normal vessels, the vascular network in tumors is chaotic, lacks hierarchy, and is characterized by irregular shape, excessive tortuosity, and non-uniform diameter. Such structural changes lead to vessels with a limited capacity to transport oxygen and nutrients [13,14]. Additionally, tumor vessels often exhibit features of immaturity, such as reduced pericyte coverage, increased permeability, and the presence of blind ends and abnormal connections. This results in the formation of shunts, increased interstitial pressure, and disturbances in blood flow, which further exacerbate hypoxia within the tumor [14,15]. Another important mechanism for the development of hypoxia is the limited diffusion of oxygen in tumor tissue. As the tumor grows, the demand for oxygen exceeds the ability of the existing vascular network to supply it, leading to the formation of an oxygen concentration gradient—from well-oxygenated areas near blood vessels to severely hypoxic regions deep within the tumor [14]. Additionally, the rapid proliferation of cancer cells causes intense oxygen consumption, which limits its availability to more distant cells. As a result, oxygen diffusion is insufficient, and the central areas of the tumor become chronically hypoxic [3]. It is also worth emphasizing that the heterogeneous and unstable blood flow in the pathological vascular network leads to uneven oxygen distribution, resulting in the occurrence of both chronic and transient hypoxia in different regions of the tumor [15]. The vascular architecture and oxygen distribution between healthy tissue and a solid tumor is preseted on Figure 1.

## 3. Strategies for Increasing Oxygen Availability in the Tumor

### 3.1. Strategies for Increasing Oxygen

Hyperbaric oxygen therapy (HBO) involves administering oxygen to the patient under increased pressure, which leads to an increase in its solubility in plasma and greater availability in tissues, including tumors. In the context of PDT, this method was studied as a way to increase oxygen levels in the tumor before or during therapy. However, the effectiveness of hyperbaric oxygenation is limited by the abnormal structure of blood vessels in the tumor and their damage caused by the therapy, which hinders the transport of oxygen to deeper areas of the cancer [16].

Improving blood flow in the tumor constitutes another strategy to increase oxygen availability. These approaches include, among others, normalization of blood vessels, reduction of interstitial pressure, and improvement of perfusion, which enables more efficient transport of oxygen to cancer cells [17]. Modulation of blood flow may also include regulation of cellular metabolism or inhibition of oxygen consumption by cancer cells, which indirectly increases its availability in the tumor microenvironment [18].

The use of vascular therapy, including vessel normalization strategies, represents a promising approach to improving tumor oxygenation. By restoring a more normal structure and function of the vessels, it is possible to increase perfusion and improve the distribution of oxygen and drugs in the tumor tissue [18]. The combination of vascular therapy with PDT or other treatment methods allows for synergistic action, enhancing the effectiveness of therapy by simultaneously improving oxygenation and increasing the sensitivity of cancer cells to treatment [12].

### 3.2. Oxygen Carriers Based on Perfluorocarbons (PFCs)

Perfluorocarbons (PFCs) are one of the most intensively studied groups of oxygen carriers in cancer therapy, particularly in the context of overcoming hypoxia and enhancing the efficacy of photodynamic therapy (PDT). These are organic compounds in which hydrogen atoms have been replaced by fluorine, giving them unique physicochemical properties favorable for gas transport [12]. One of the key characteristics of perfluorocarbons is their exceptionally high capacity to dissolve gases, including oxygen and carbon dioxide. This property results from weak intermolecular interactions and a chemical structure rich in C–F bonds, which enables effective storage and transport of oxygen to hypoxic areas within the tumor [19]. Thanks to this, PFCs can act as “oxygen reservoirs,” increasing its local concentration in the tumor microenvironment and improving the effectiveness of oxygen-dependent therapies, such as PDT [20]. Perfluorocarbons are chemically and biologically inert, which means that they do not easily undergo metabolic reactions or degradation in the body. This stability makes them safe carriers in biomedical applications and allows them to be used as components of nanocarriers without significant risk of side reactions [19]. Additionally, their bioinertness and the ability to be minimally metabolized enable their use in drug and gas delivery systems without significant interference with the body’s biological processes [21].

PFC nanoemulsions are among the most commonly used forms of oxygen carriers, stabilized by lipids, polymers, or surfactants. Their small size enables effective distribution in the body and accumulation in the tumor through the enhanced permeability and retention (EPR) effect [19]. Nanoemulsions can simultaneously transport oxygen and photosensitizers, which allows for a synergistic increase in the effectiveness of photodynamic therapy under hypoxic conditions [22].

Nanodroplets of PFC represent an advanced form of carriers, which can be activated by external stimuli, such as light or ultrasound. Upon activation, they can undergo a phase transition (from liquid to gas), forming microbubbles and enabling controlled oxygen release directly at the tumor site [19]. Studies have shown that nanodroplets can significantly increase oxygen levels in tumor tissue, even several times over, which translates into improved effectiveness of anticancer therapies [22].

PFC-stabilized microbubbles constitute an alternative form of oxygen carriers, used both in diagnostics and therapy. They can transport significant amounts of gases and be controllably activated, which allows their use in imaging as well as targeted therapy. Their ability to transport oxygen and interact with ultrasound makes them promising tools in cancer therapy, particularly in the context of improving tumor oxygenation [23].

Perfluorocarbons act as oxygen carriers by physically dissolving and transporting it in the bloodstream and then releasing it in areas with lower oxygen partial pressure, such as tumor tissues. Due to accumulation in the tumor, they can locally increase oxygen availability and counteract hypoxia [19]. Modern nanodroplet systems also enable controlled, spatio-temporal oxygen release, which increases therapy precision and limits side effects [22]. An important mechanism for improving the effectiveness of photodynamic therapy using PFC is the prolongation of the singlet oxygen (^1^O_2_) lifetime. In a perfluorocarbon environment, the lifetime of this reactive oxygen species is significantly longer than in an aqueous or cellular environment, which increases the efficiency of oxidative reactions and leads to a stronger cytotoxic effect against cancer cells [16]. This phenomenon, combined with a high capacity for oxygen storage, makes PFCs significantly enhance ROS generation and the effectiveness of PDT even under hypoxic conditions [22].

#### 3.2.1. PFC as a Theranostic Element

Perfluorocarbons (PFCs) are widely used as components of theranostic systems, which combine therapeutic and diagnostic functions. One of the most important directions is their integration with photosensitizers to enhance the efficacy of photodynamic therapy (PDT). PFC nanodroplets can simultaneously transport oxygen and a photosensitizer to the tumor, which allows their colocalization and significantly increases the efficiency of reactive oxygen species (ROS) generation. This approach enables overcoming the limitations caused by hypoxia by simultaneously delivering the substrate (oxygen) and the active agent (photosensitizer). Additionally, the use of PFC in theranostic nanostructures allows controlled, light-induced release of oxygen and the photosensitizer at the tumor site, which increases the precision of action and limits therapy side effects [22]. PFCs can also be used as multifunctional carriers, capable of co-transporting oxygen and anticancer drugs, which allows for a synergistic therapeutic effect. By improving tumor oxygenation, the effectiveness is increased not only for PDT but also for other therapies, such as chemotherapy or radiotherapy, whose efficacy is strongly dependent on the presence of oxygen [22]. In theranostic systems, PFCs enable the simultaneous delivery of therapeutic components and monitoring of treatment effects, which represents an important step toward personalized medicine [23]. An important aspect of PFC-based theranostics is their ability to integrate with imaging techniques, such as ultrasonography and photoacoustic imaging. PFC nanodroplets can contain contrast agents (e.g., indocyanine green–ICG), which allow monitoring of the carrier distribution, oxygenation levels, and therapy effectiveness in real time [22]. Thanks to this, it is possible to conduct image-guided therapy, in which oxygen delivery and photosensitizer activation are precisely controlled spatially and temporally [23].

#### 3.2.2. Stimuli-Activated Systems

Systems based on PFC can be activated by external stimuli, such as ultrasound, which allows controlled oxygen release directly within the tumor. In the case of microbubbles containing PFC, the application of ultrasound leads to their destruction (ultrasound-targeted microbubble destruction), resulting in a rapid release of oxygen and an increase in its local concentration. Studies have shown that such an approach can significantly improve tumor oxygenation and increase singlet oxygen production during PDT, which translates into higher therapeutic efficacy and inhibition of tumor progression [24]. Advanced PFC systems utilize the phase transition phenomenon, in which nanodroplets (liquid) can be transformed into microbubbles (gas) under the influence of stimuli such as ultrasound or light energy. This process, referred to as acoustic or optical vaporization, enables precise oxygen release while simultaneously enhancing imaging contrast. This conversion not only increases the efficiency of oxygen delivery but also improves the penetration and retention of carriers in tumor tissue, making it a particularly promising strategy in the therapy of hypoxic tumors [21].

### 3.3. Nanocarriers Simultaneously Delivering Oxygen and Photosensitizer

The development of nanotechnology has enabled the creation of advanced therapeutic systems that simultaneously deliver oxygen and a photosensitizer to the tumor, which allows overcoming one of the main limitations of photodynamic therapy (PDT), namely hypoxia. Such nanocarriers increase the local oxygen concentration while also ensuring the presence of the active agent, leading to more effective generation of reactive oxygen species (ROS) and enhancement of the anticancer effect [16,25]. One approach is the use of liposomes containing hemoglobin as a natural oxygen carrier along with a photosensitizer. Hemoglobin, due to its ability to bind oxygen, enables its transport and controlled release within the tumor, which increases the effectiveness of PDT. Research on biomimetic nanocarriers has shown that such systems can significantly improve the oxygenation of the tumor microenvironment and increase ROS production during therapy. Additionally, the integration of hemoglobin with a photosensitizer in a single nanoscale system allows their co-localization in cancer cells, which enhances the efficiency of photodynamic reactions and leads to a stronger cytotoxic effect [25]. An alternative and widely studied strategy is the use of nanocapsules containing perfluorocarbons (PFCs) and photosensitizers. PFCs, due to their high oxygen solubility, act as oxygen reservoirs, while the photosensitizer enables ROS generation upon light activation. In such systems, the oxygen stored in the PFC core is directly used in photodynamic reactions, which significantly increases the efficiency of singlet oxygen production [16,20]. Nanocapsules of this type can additionally serve a theranostic function, enabling simultaneous imaging (e.g., ultrasonographic) and therapy, which allows for monitoring the effects of treatment in real time [20].

#### 3.3.1. Examples

An example of a biomimetic system is a nanoplatform containing oxygen-transporting components (e.g., hemoglobin or its analogs) and indocyanine green (ICG) as a photosensitizer. Studies have shown that such systems increase ROS production and improve the effectiveness of photodynamic therapy by simultaneously delivering oxygen and the active therapeutic agent. Additionally, the use of ICG enables fluorescence imaging, which allows tracking of the nanocarrier distribution and optimization of the tumor irradiation timing [25]. One of the best-described examples is a system based on perfluorooctylbromide (PFOB) and the photosensitizer IR780. Studies have shown that nanocapsules containing these components increase the oxygen concentration in the tumor, leading to more intense singlet oxygen production during PDT and more effective destruction of cancer cells [20]. Additionally, IR780 exhibits mitochondrial targeting capability, which increases the efficiency of therapy by directly damaging key cellular structures [26].

#### 3.3.2. Biological Effects

Increasing oxygen availability in the tumor microenvironment through the use of nanocarriers leads to a reduction in the stabilization of the HIF-1α factor, which is a key regulator of the hypoxia response. Studies have shown that improving tumor oxygenation can reverse hypoxic signaling pathways and limit the adaptation of cancer cells to oxygen deprivation [27]. Nanocarriers that simultaneously deliver oxygen and a photosensitizer exhibit a pronounced anticancer effect, leading to the inhibition of tumor growth in in vivo models. Increased ROS production and improved oxygenation enhance apoptosis of cancer cells and limit disease progression [14,24]. Preclinical studies have shown that the use of such nanoplatforms results in a significant improvement in the survival of animal models, which is due to more effective destruction of cancer cells and reduction of hypoxia. The combination of oxygen delivery and photodynamic therapy therefore constitutes a promising therapeutic strategy [27].

### 3.4. “Self-Oxygen Supply” Strategies

“Self-oxygen supply” strategies (self-delivery of oxygen) represent a modern approach to overcoming tumor hypoxia, involving the design of systems capable of locally storing or generating oxygen without the need for external delivery. This approach is particularly significant in photodynamic therapy (PDT), where effectiveness directly depends on the availability of oxygen in the tumor microenvironment [19]. One of the most innovative solutions is fluorinated photosensitizers, which, due to the presence of fluorine atoms, exhibit an increased ability to dissolve and store oxygen. In such systems, the photosensitizer serves a dual function—both as a reactive oxygen species (ROS)-generating agent and as a local oxygen reservoir, which allows for increased PDT effectiveness under hypoxic conditions. Fluorinated systems can transport oxygen to the tumor site due to their high gas solubility and then release it in response to tumor microenvironment conditions, such as low pH or the presence of specific enzymes. This makes it possible to increase local oxygen availability and improve the efficiency of singlet oxygen generation during therapy. Additionally, the use of fluorinated structures reduces photosensitizer aggregation and improves their photophysical properties, leading to increased production of reactive oxygen species and a stronger cytotoxic effect on cancer cells [28]. Another approach within the “self-oxygen supply” strategy is the use of nanomaterials capable of storing or generating oxygen directly at the tumor site. This group includes, among others, nanomaterials based on metal–organic frameworks (MOFs), nanocatalysts, and enzymatic systems that can convert endogenous molecules (e.g., H_2_O_2_) into oxygen [27]. These nanomaterials enable in situ oxygen generation, which allows therapy to be independent of the existing oxygenation level in the tumor. This makes it possible to maintain continuous ROS production even in strongly hypoxic areas [24]. In many cases, these nanomaterials are designed as multifunctional systems that combine the ability to generate oxygen with the delivery of photosensitizers and imaging capabilities, which increases their clinical potential [19].

## 4. Alternative Approach: Type I Photosensitizers

The development of type I photosensitizers represents one of the most important alternative approaches to classical photodynamic therapy (PDT), especially in the context of treating tumors with low oxygen levels. Unlike traditional photosensitizers, which require the presence of molecular oxygen, type I mechanisms allow for the generation of reactive oxygen species (ROS) even under hypoxic conditions, which significantly increases the therapeutic potential of this method [29].

### 4.1. Differences Between Type I and Type II PDT

Classical photodynamic therapy is mainly based on the type II mechanism, in which the excited photosensitizer transfers energy directly to an oxygen molecule (O_2_), leading to the formation of singlet oxygen (^1^O_2_). This highly reactive species is the main cytotoxic agent responsible for damage to biomolecules such as lipids, proteins, and DNA, ultimately leading to cell death [30]. However, this mechanism is highly dependent on the availability of oxygen in the tissue, which poses a significant limitation in the case of hypoxic tumors, where low oxygen concentration significantly reduces the efficiency of ^1^O_2_ generation and thus the effectiveness of the therapy [31]. In contrast to type II, the type I mechanism relies on redox reactions, in which the excited photosensitizer participates in the transfer of electrons or hydrogen atoms to surrounding molecules. This process leads to the formation of free radicals and secondary reactive oxygen species, which can damage cellular structures even with limited oxygen availability [32]. Type I photosensitizers therefore show less dependence on oxygen and are particularly promising in the treatment of tumors with a strongly hypoxic microenvironment, where traditional type II approaches are less effective [31].

### 4.2. Mechanism of Action of Type I Photosensitizers

The mechanism of action of type I photosensitizers begins with the absorption of light and the transition of the molecule to the excited triplet state. In this state, the photosensitizer can engage in redox reactions with surrounding substrates, either donating or accepting electrons. This process leads to the formation of reactive radicals and initiates a cascade of chemical reactions that result in cell damage [33]. Electron transfer can occur both with the involvement of biological molecules (e.g., lipids or proteins) and dissolved oxygen, leading to the formation of secondary reactive oxygen species. Unlike the type II mechanism, this process does not require the direct transfer of energy to molecular oxygen, making it more versatile under hypoxic conditions [32]. One of the main products of type I reactions are hydroxyl radicals (•OH), which are among the most reactive forms of oxygen and exhibit strong cytotoxic properties. They are formed as a result of secondary reactions, such as the disproportionation of the superoxide anion radical or Fenton-type reactions. Hydroxyl radicals can rapidly react with biomolecules, leading to their irreversible damage [29]. Their short lifespan and high reactivity make them act locally, which increases the selectivity of damage within cancer cells [34]. Another important product of type I reactions is the superoxide anion radical (O_2_•^−^), formed as a result of the reduction of an oxygen molecule by an excited photosensitizer. Although it is less reactive than the hydroxyl radical itself, it plays a key role as a precursor of other reactive oxygen species [29]. The superoxide anion radical can undergo further transformations, leading to the formation of hydrogen peroxide and hydroxyl radicals, which enhances the cytotoxic effect and allows for the effective destruction of cancer cells even under conditions of limited oxygen availability [31].

### 4.3. Design of Type I Photosensitizers

The design of type I photosensitizers focuses on optimizing their ability to generate reactive oxygen species (ROS) under limited oxygen availability and on improving their photophysical and biological properties. In recent years, particular emphasis has been placed on strategies that increase the efficiency of radical reactions, control aggregation, and utilize advanced nanostructures [27]. One of the key goals in designing type I photosensitizers is to enhance ROS generation efficiency under hypoxic conditions. Unlike classical type II photosensitizers, which require oxygen to produce singlet oxygen, type I systems are designed to favor electron transfer reactions and generate free radicals even at low oxygen concentrations. Research on modern porphyrins and their derivatives has shown that appropriate modification of the chemical structure can significantly increase the contribution of the type I mechanism, leading to effective destruction of cancer cells in a hypoxic environment [35]. Additionally, photosensitizers capable of simultaneous activation of alternative cell death mechanisms (e.g., ferroptosis) are being developed, which may lead to increased intracellular oxygen availability and further enhance the therapeutic effect [36]. An important direction in designing type I photosensitizers is the use of the aggregation phenomenon, which in many modern systems leads to increased ROS generation efficiency. Unlike classical photosensitizers, which undergo quenching in the aggregated state (ACQ), new materials, such as photosensitizers with AIE (aggregation-induced emission) properties, exhibit increased photodynamic activity upon aggregation. Studies have shown that aggregation can promote intersystem crossing processes between singlet and triplet states and enhance electron transfer efficiency, which directly translates into more intense ROS production via the type I mechanism. This approach allows for the design of photosensitizers with high activity even under the challenging conditions of the tumor microenvironment, where traditional systems lose their effectiveness [37]. Nanostructures play a key role in designing modern type I photosensitizers, as they enable control over their physicochemical properties, bioavailability, and selectivity toward cancer cells. Nanoparticles can be designed to increase the stability of the photosensitizer, improve its solubility, and allow its accumulation in the tumor through the EPR effect. Additionally, the use of nanostructures enables the integration of photosensitizers with additional functions, such as in situ oxygen generation, drug delivery, or imaging, which significantly enhances the efficacy of therapy [19]. Modern nanoplatforms often utilize donor–acceptor (D–A) structures that promote charge separation and increase the efficiency of redox reactions, which directly translates into more effective ROS generation via the type I mechanism [37].

### 4.4. Photosensitizers Activating the Immune System

The combination of photodynamic therapy with immunotherapy represents a promising direction in cancer treatment, particularly in the context of type I photosensitizers. Reactive oxygen species generated during PDT can induce immunogenic cell death (ICD), leading to the release of tumor antigens and activation of the immune response. Type I photosensitizers are especially useful in this context because they can act effectively under hypoxic conditions, which often limit the effectiveness of classical PDT and the immune response. Studies have shown that therapy combining PDT and immunotherapy can lead to T lymphocyte activation and enhancement of the antitumor response, which translates into better therapeutic effects and the potential elimination of metastases [37]. One of the most important immunological mechanisms associated with type I photosensitizers is their ability to modulate macrophage activity in the tumor microenvironment. In particular, it has been shown that type I photosensitizers can transform M0 and M2 (pro-tumor) macrophages into the M1 phenotype with antitumor activity. This mechanism is associated with the generation of ROS, which activate signaling pathways such as NF-κB, leading to the production of pro-inflammatory cytokines and an increase in the phagocytic capacity of macrophages. Activated M1 macrophages can then induce cancer cell death, support antigen presentation, and stimulate the adaptive response through T lymphocyte activation, resulting in a long-lasting antitumor effect [37].

## 5. Combined Therapies Using Hypoxia

Combined therapies are one of the most promising approaches in cancer treatment, especially in the context of hypoxia, which limits the effectiveness of photodynamic therapy (PDT). Using hypoxia as a therapeutic element—rather than solely an obstacle—allows for the design of strategies in which oxygen deprivation activates additional cytotoxic mechanisms, leading to a synergistic anticancer effect [38].

### 5.1. PDT + Hypoxia-Activated Prodrugs

**Figure 2:**
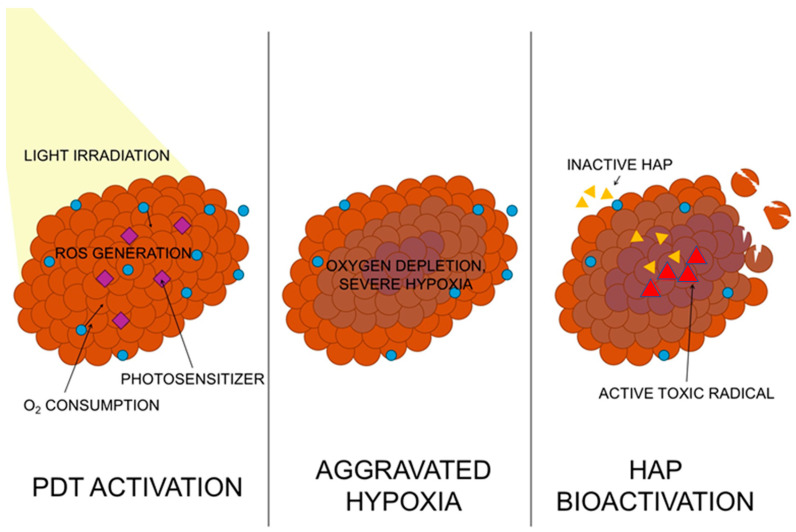
Schematic representation of the synergistic anticancer effect combining photodynamic therapy (PDT) and hypoxia-activated prodrugs (HAPs). The therapy follows a three-step cascade: (Step 1) Light irradiation activates the photosensitizer (PS), leading to the generation of reactive oxygen species (ROS), which induce direct cytotoxicity and rapidly consume local molecular oxygen (O_2_). (Step 2) This oxygen consumption, combined with the tumor’s poor vasculature, leads to a state of aggravated or severe hypoxia. (Step 3) The low-oxygen environment triggers the bioactivation of the inactive prodrug (represented by yellow triangles) into highly toxic radicals (red triangles). These radicals effectively eliminate cancer cells in deep, hypoxic regions that are typically resistant to conventional oxygen-dependent therapies. The blue color means oxygen.

One of the best-known approaches is the combination of PDT with hypoxia-activated prodrugs (HAPs), such as tirapazamine (TPZ). These prodrugs are selectively activated under low oxygen conditions, where they undergo enzymatic reduction and transformation into cytotoxic forms capable of damaging the DNA of cancer cells [39]. In the context of combination therapy, PDT plays a dual role: on one hand, it generates ROS and directly destroys cancer cells, and on the other hand, it consumes available oxygen, deepening hypoxia in the tumor. The resulting conditions favor the activation of tirapazamine, leading to an additional cytotoxic effect in hypoxic areas. Studies have shown that the combination of PDT (e.g., based on 5-aminolevulinic acid) with tirapazamine leads to increased apoptosis, inhibition of cancer cell proliferation, and a stronger anticancer effect than each method applied separately [40]. In cancer models, it has also been demonstrated that combination therapy leads to tumor growth inhibition and an increase in necrotic areas, indicating a synergistic effect of both therapy components [41]. Additionally, modern nanocarrier systems enable the simultaneous delivery of a photosensitizer and tirapazamine to the tumor (Figure 2, allowing precise control of therapy and increasing its effectiveness through the activation of the prodrug directly in the hypoxic microenvironment [42].

### 5.2. PDT + Chemotherapy

The combination of photodynamic therapy with classical chemotherapy constitutes another important therapeutic strategy that allows for increased treatment effectiveness by utilizing different mechanisms of action. PDT induces oxidative stress and cellular damage, while chemotherapy acts by disrupting cell division or directly damaging DNA [38]. In combined systems, nanocarriers capable of simultaneously delivering a photosensitizer and a chemotherapeutic drug are often used, which allows for their colocalization in the tumor and enhances the therapeutic effect. This approach also allows for the reduction of cytotoxic drug doses and the limitation of side effects. In particular, it has been shown that PDT can induce hypoxia, which subsequently activates environment-dependent drugs (e.g., prodrugs), leading to a cascade effect and increased efficacy of anticancer therapy [43]. Additionally, the combination of PDT with chemotherapy can overcome tumor resistance to treatment by simultaneously affecting various biological pathways, making it more difficult for cancer cells to adapt to therapy [38].

### 5.3. PDT + Immunotherapy

The combination of photodynamic therapy with immunotherapy is one of the most dynamically developing directions in oncology. PDT, through the generation of ROS, can induce immunogenic cell death (ICD), which leads to the release of tumor antigens and activation of the immune system [38]. Studies indicate that PDT can enhance the effectiveness of immune checkpoint inhibitors (e.g., PD-1/PD-L1), leading to a stronger antitumor response. PDT affects the tumor microenvironment by modulating the expression of factors related to hypoxia (e.g., HIF-1α), which play a significant role in regulating the immune response and therapy efficacy [44]. The combination of PDT with immunotherapy enables not only local tumor destruction but also the induction of a systemic response, which can lead to the elimination of metastases and long-term disease control [38].

## 6. Comparison of Strategies

### 6.1. Oxygen Delivery vs. Oxygen Independence

**Figure 3:**
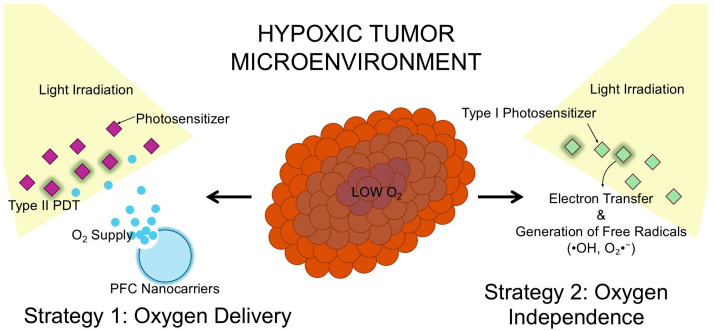
Schematic comparison of the two principal strategies for overcoming tumor hypoxia in photodynamic therapy (PDT).

Contemporary strategies for counteracting tumor hypoxia can be divided into two main approaches: (1) increasing the availability of oxygen in the tumor and (2) developing therapeutic methods less dependent on its presence, such as type I photosensitizers. Both approaches arise from a fundamental limitation of anticancer therapies, whose effectiveness depends on the generation of reactive oxygen species (ROS) [45]. Oxygen delivery-based strategies, such as the use of perfluorocarbons (PFCs), focus on increasing the local concentration of O_2_ in the tumor microenvironment (Figure 3). PFCs act as carriers of physically dissolved oxygen, which can be transported and released in hypoxic areas, directly enhancing the effectiveness of oxygen-dependent therapies, such as PDT [46]. On the other hand, oxygen-independent approaches, such as type I photosensitizers, utilize radical mechanisms based on electron transfer, which can occur even at low oxygen concentrations. This makes it possible to generate ROS under hypoxic conditions, making this strategy particularly attractive for tumors with severe hypoxia [29]. Comparing both approaches, it can be observed that oxygen delivery strategies aim to “repair” the tumor microenvironment by oxygenating it, whereas type I photosensitizers bypass this problem by adapting their mechanism of action to the existing biological conditions [19].

The central area represents the severely hypoxic tumor microenvironment characterized by low oxygen availability (low O_2_), which critically limits conventional treatments. To counteract this limitation, two distinct approaches are employed. Left (Strategy 1: Oxygen Delivery): Nanocarriers such as perfluorocarbons (PFCs) or hemoglobin analogs actively transport and release oxygen within the tumor bed. This local oxygenation restores the efficacy of the classical type II PDT mechanism, leading to the efficient generation of singlet oxygen (^1^O_2_). Right (Strategy 2: Oxygen Independence): An alternative, hypoxia-tolerant approach utilizes type I photosensitizers. Upon light irradiation, these molecules bypass the need for molecular oxygen by undergoing direct electron transfer reactions with adjacent substrates, generating highly cytotoxic free radicals, such as hydroxyl radicals (•OH) and superoxide anions (O_2_•^−^), to effectively eradicate cancer cells.

### 6.2. Advantages and Limitations

One of the greatest advantages of perfluorocarbons is their exceptionally high capacity to dissolve gases, including oxygen, which far exceeds solubility in water or plasma. As a result, PFCs can act as effective oxygen reservoirs, enabling its transport to hypoxic areas and improving the efficacy of anticancer therapies [46]. Additionally, the ability to prolong the singlet oxygen lifetime in a PFC environment increases the efficiency of photodynamic reactions and enhances the therapeutic effect [45]. Despite numerous advantages, PFC-based systems are characterized by significant technological complexity. Due to the hydrophobic and lipophobic nature of these compounds, it is necessary to create stable nanoemulsions or other advanced delivery forms, which hinders their development and clinical application [46]. Optimizing the pharmacokinetics, stability, and safety of such systems poses a significant challenge that limits their widespread use in clinical practice [47]. A key advantage of type I photosensitizers is their ability to act under low oxygen concentration conditions. Electron transfer-based mechanisms allow for the generation of ROS independently of molecular oxygen availability, which significantly increases the effectiveness of therapy in the hypoxic tumor microenvironment [29]. This approach thus eliminates one of the main barriers of classical PDT and allows for effective destruction of cancer cells even in deeply hypoxic tumor regions [19]. One of the potential limitations of type I photosensitizers is their lower selectivity of action compared to the type II mechanism. Radicals generated in type I reactions, such as hydroxyl radicals or superoxide anion radicals, are highly reactive and can interact with a wide range of biomolecules, which increases the risk of damage to healthy tissues [29]. Moreover, difficulties in precisely controlling the location and intensity of ROS generation may limit therapy safety and require further research on improving targeting and controlling photosensitizer activity [19]. In summary, oxygen delivery strategies and oxygen-independent approaches represent two complementary directions in the development of anticancer therapies. While PFCs offer the possibility of improving tumor oxygenation and enhancing the efficacy of oxygen-dependent therapies, type I photosensitizers allow bypassing the hypoxia problem by utilizing alternative ROS generation mechanisms. Optimal future therapeutic approaches will likely combine both of these mechanisms to maximize treatment efficacy.

## 7. Challenges and Limitations

Despite the dynamic development of strategies to counteract tumor hypoxia, their implementation in clinical practice encounters a number of significant limitations. These issues concern both the properties of the nanocarriers themselves and their interactions with the body, control of their activity, and the translation of preclinical research results into clinical applications [48]. One of the key challenges associated with the use of nanocarriers in cancer therapy is their potential toxicity. Due to their small size and large surface area, nanoparticles can interact with biomolecules and cellular structures, which can lead to oxidative stress, cellular damage, and inflammatory reactions [49]. Particular attention is paid to the accumulation of nanoparticles in organs such as the liver and spleen, where they may be captured by the reticuloendothelial system (RES). Prolonged retention in these organs may lead to toxic effects, including hepatotoxicity and immune system dysfunction [50]. The physicochemical properties of nanoparticles, such as size, shape, surface charge, and chemical composition, have a significant impact on their toxicological profile, which requires careful optimization at the design stage [51]. Another important limitation is the low bioavailability of nanocarriers within the tumor and the complex pharmacokinetics of their distribution. After administration into the body, nanoparticles undergo processes of absorption, distribution, metabolism, and excretion (ADME), which determine their therapeutic effectiveness [48]. Studies have shown that only a small percentage of the administered nanoparticle dose reaches the tumor—on average below a few percent of the total dose—which constitutes a serious limitation to the effectiveness of therapy [52]. Most nanoparticles accumulate in filtering organs such as the liver and spleen, which limits their availability at the target site and may increase the risk of adverse effects [48]. Another significant problem is the limited penetration of nanoparticles into the depth of tumor tissue—many of them accumulate mainly near blood vessels, which hinders reaching cells located in more distant, highly hypoxic areas of the tumor [51]. The effectiveness of oxygen-based delivery strategies depends not only on its amount but also on precise control over the location and timing of release. One of the main challenges is ensuring that oxygen is released exactly in the tumor area and at the right time to maximize the effectiveness of therapies such as PDT [45]. Lack of control over oxygen release can lead to its premature diffusion or loss in systemic circulation, which significantly limits the effectiveness of therapy [46]. The variability of the tumor microenvironment (e.g., differences in perfusion, pH, or interstitial pressure) makes it difficult to predict and control the behavior of oxygen-delivery systems, which constitutes a significant challenge in designing effective therapies [19]. Despite promising results from preclinical studies, the translation of nanotechnology into clinical practice remains limited. Many systems show high effectiveness in in vitro and in vivo (animal) models, but their efficiency in clinical trials is significantly lower [48]. One of the main reasons is the difference between experimental models and the actual microenvironment of human tumors, which is characterized by greater heterogeneity and a more complex structure [53]. Additionally, issues related to safety, production scalability, formulation stability, and legal regulations constitute significant barriers in the process of implementing new therapies. Consequently, despite intensive research on nanocarriers and strategies to counteract hypoxia, only a small number of systems have reached the stage of clinical applications, highlighting the need for further studies on the optimization of their properties and a better understanding of their interactions with the body [48].

## 8. Prospects for Future Research

The dynamic development of nanomedicine and photodynamic therapy is leading to the creation of increasingly advanced cancer treatment strategies, particularly in the context of overcoming hypoxia. Contemporary research directions focus on increasing the precision of therapy, integrating different therapeutic approaches, and tailoring treatment to the individual characteristics of the patient and the tumor [54]. The development of intelligent nanocarriers responsive to stimuli is one of the most promising directions for future research in nanomedicine. These systems enable controlled drug release in response to specific tumor microenvironment conditions, such as changes in pH, the presence of enzymes, redox potential, or levels of reactive oxygen species. This makes it possible to limit premature drug release in the systemic circulation and increase its concentration at the target site, which translates into higher therapeutic efficacy and lower toxicity. In the future, designing multifunctional nanoplatforms capable of responding to multiple stimuli simultaneously and integrating therapeutic and diagnostic functions (so-called theranostics) will be key [55]. The further development of this technology will include optimizing the pharmacokinetic properties of nanocarriers, such as circulation time, bioavailability, and tumor penetration ability. Current research indicates that the use of both internal stimuli (e.g., hypoxia, ROS) and external stimuli (light, magnetic field, ultrasound) allows for precise control of drug release and increased selectivity of anticancer therapy [56]. The integration of nanotechnology with cancer immunotherapy represents another key research direction. Nanocarriers can be used to deliver immunomodulators, tumor antigens, or immune checkpoint inhibitors in a spatially and temporally controlled manner. Particularly promising are light-sensitive systems, which allow activation of the immune response at the tumor site through the induction of immunogenic cell death of cancer cells [57]. Research also indicates that ROS-responsive nanocarriers can significantly enhance the effect of photodynamic immunotherapy by increasing the production of reactive oxygen species and activating immune system cells such as T lymphocytes or dendritic cells. This approach leads to a synergistic therapeutic effect, combining direct destruction of cancer cells with a long-lasting immune response [58]. Therapy personalization based on nanocarriers represents the future of cancer treatment, enabling the adaptation of therapeutic strategies to the individual characteristics of the patient and the specificity of the tumor microenvironment. Nanocarriers can be designed to respond to the unique biomarkers of a particular cancer, allowing for selective drug delivery and minimization of side effects. In the future, the development of this field will be based on integrating molecular data (e.g., genetic and proteomic profiles) with the design of therapeutic nanoplatforms. This approach will enable the creation of highly precise systems capable of adapting to dynamic changes in the tumor microenvironment and increasing treatment efficacy through therapy individualization [59]. One of the biggest limitations of photodynamic therapy (PDT) is hypoxia occurring in the tumor microenvironment, which significantly reduces the efficiency of reactive oxygen species generation. In response to this problem, hybrid nanomedicine systems capable of simultaneously delivering oxygen and inducing type I PDT reactions, which are less dependent on molecular oxygen, are being developed. Modern nanoplatforms are designed to alleviate hypoxia by delivering oxygen or catalytically generating it at the tumor site, while simultaneously increasing ROS production through type I mechanisms. The integration of these strategies leads to a significant increase in therapy effectiveness and enables overcoming the limitations of classical PDT [54]. Furthermore, the development of hybrid nanomedicines involves combining photodynamic therapy with other methods, such as photothermal therapy or immunotherapy, which allows for achieving a synergistic effect and increasing the effectiveness of cancer treatment. Such multifunctional systems, responsive to the tumor microenvironment, represent the future of integrated therapeutic strategies [60,61].

Multi-Energy Integrated Photocatalysis for Antitumor Therapy is using nanotheranostic systems that combine piezo-photocatalysts and photothermal-photocatalysts for improved cancer treatment. These systems generate cytotoxic reactive oxygen species (ROS) by integrating heat or mechanical energy with light-driven reactions. The approach overcomes conventional photodynamic therapy limitations, such as low tissue penetration and hypoxia. It also maps the development of complex, multi-modal therapies combining photocatalysis with immunotherapy and chemotherapy [62]. An “all-in-one” nanoplatform was developed to enhance cancer immunotherapy by combining photo-immunotherapy with immune checkpoint blockade to convert “cold” tumors into “hot” ones. Under mild near-infrared laser light, the system induces immunogenic cell death and boosts cytotoxic T cell infiltration, enhancing the efficacy of the anti-PD-L1 antibody [63].

The development of the nanoreactor, which integrates chlorin e6, glucose oxidase, ZIF-8, polydopamine, and manganese dioxide via a one-pot synthesis to address tumor hypoxia and glutathione overproduction, was designed. Inside tumor cells, this cascade bioreactor triggers degradation to simultaneously enable photodynamic, chemodynamic, and starvation therapies [64].

## 9. Conclusions

Hypoxia constitutes one of the key barriers limiting the effectiveness of cancer therapies, particularly photodynamic therapy (PDT). Low oxygen concentration in the tumor microenvironment results from impaired perfusion and high oxygen consumption by cancer cells, which leads to a significant reduction in the efficiency of reactive oxygen species (ROS) generation, which is necessary for PDT action. Additionally, photodynamic therapy itself can exacerbate hypoxia through further consumption of available oxygen, creating a vicious cycle that limits treatment effectiveness [18,38]. Among the most promising strategies to overcome hypoxia, oxygen delivery systems, especially those based on perfluorocarbons (PFCs), hold particular importance. These compounds are characterized by an exceptionally high capacity for dissolving oxygen, which allows for its effective transport and release within the tumor. Studies have shown that PFC nanocarriers increase local oxygen concentration, enhance singlet oxygen generation, and lead to significant inhibition of tumor growth compared to conventional PDT [18,63]. In parallel, type I photosensitizers are being developed, which can act effectively even under conditions of limited oxygen availability. Unlike classical type II mechanisms, type I photosensitizers generate free radicals through electron transfer reactions, allowing the initiation of cytotoxic processes at lower oxygen concentrations. Therefore, they constitute an important alternative to traditional PDT strategies and enable more effective treatment of hypoxic tumors [65]. The future of anticancer therapies aimed at overcoming hypoxia lies in the development of hybrid approaches and combination therapies. Combining strategies for oxygen delivery (e.g., using PFC or hemoglobin) with the simultaneous use of photosensitizers or other therapeutic methods (e.g., chemotherapy or metabolism inhibitors) allows for a synergistic increase in treatment effectiveness. Such multifunctional systems can simultaneously improve tumor oxygenation, limit oxygen consumption, and increase ROS production, making them one of the most promising directions for further research [42,66,67].

## Figures and Tables

**Table 1:** Comparative analysis of oxygen-delivery platforms and type I photosensitizers for overcoming tumor hypoxia.

Feature/Criteria	Physical Oxygen-Delivery Carriers (O_2_-Transport)	Chemical Type I Photosensitizers (O_2_-Independent PDT)
Primary Mechanism	Physically transport and release molecular oxygen O_2_ directly into the hypoxic tumor core.	Generate toxic free radicals (via electron transfer) utilizing ambient substrates instead of O_2_.
Common Nanoparticle Platforms	Liposomes and Polymeric Micelles (encapsulating PFCs/Hb) Metal–Organic Frameworks (MOFs) (with high gas-storage capacity) Mesoporous Silica Nanoparticles (MSNs) Albumin Nanoparticles (e.g., HSA-stabilized Hb)	Aggregation-Induced Emission (AIE) Dots Semiconductor Quantum Dots (e.g., CdSe, ZnS) Carbon Dots and Graphene Oxide Upconversion Nanoparticles (UCNPs) (coupled with type I dyes) Polymer Nanoparticles (encapsulating hypoxia-tolerant dyes)
Reactive Species Generated	Singlet oxygen ^1^O_2_ via standard type II photochemical pathways.	Hydroxyl radicals, superoxide anions O_2_^−^, and hydrogen peroxide H_2_O_2_.
Dependence on Tissue O_2_	High (Relies on successful oxygen loading, transport, and local release).	Low to None (Functions effectively in deeply hypoxic or near-anoxic environments).
Key Materials & Examples	Perfluorocarbons (PFCs), hemoglobin-based oxygen carriers (HBOCs), hyperbaric O_2_ microbubbles.	Modified porphyrins, titanium dioxide (TiO_2)_ nanoparticles, organic radical precursors (e.g., iodinated dyes).
Major Advantages	• Replenishes local O_2_ levels • Reverses hypoxia-mediated radiation resistance • Normalizes tumor microenvironment	• No external O_2_ source required • Highly efficient in severe hypoxia • Less prone to oxygen consumption depletion
Key Limitations	• Limited payload capacity • Risk of premature O_2_ leakage during circulation • High dependency on local blood flow	• Complex chemical synthesis • Potential dark toxicity of free radical precursors • Lower quantum yield compared to classical type II
Biocompatibility Concerns	Potential reticuloendothelial system (RES) accumulation and long-term tissue retention.	Potential off-target cellular toxicity due to highly reactive free radical generation.
Future Clinical Outlook	Ideal for combination with radiotherapy or traditional type II PDT to maximize oxygenation.	Ideal for treating deeply buried, necrotic, or severely hypoxic solid tumor cores.

## Data Availability

No new data were created.
